# Investigating fucoidan blend supplementation and resistance training in humans: a parallel randomized controlled trial design

**DOI:** 10.1038/s41598-025-24066-9

**Published:** 2025-11-17

**Authors:** Stephen D. Cousins, Samantha T.C Kucewicz, Daniel W.T Wundersitz, Blake E.G. Collins, Matthew B. Cooke, Nicola McKeown, Hericka B. Figueiredo Galvao, Stephanie M. Resciniti, Phil M. Lyristakis, Brett A. Gordon, Chris van der Poel

**Affiliations:** 1 Department of Microbiology, Anatomy, Physiology, and Pharmacology, School of Life Sciences, La Trobe University, Bundoora, VIC Australia; 2 Holsworth Biomedical Research Centre, La Trobe Rural Health School, La Trobe University, Bendigo, VIC Australia; 3 Sport, Performance, and Nutrition Research Group, School of Allied Health, Human Services, and Sport, La Trobe University, Melbourne, VIC Australia; 4 Department of Food, Nutrition and Dietetics, School of Allied Health, Human Services and Sport, La Trobe University, Bundoora, VIC Australia; 5 Centre for Cardiovascular Biology and Disease Research (CCBDR), La Trobe Institute of Medical Science (LIMS), La Trobe University, Melbourne, VIC Australia

**Keywords:** Fucoidan, Supplementation, Resistance training, Anaerobic metabolism, Body composition, Metabolism, Translational research

## Abstract

Fucoidan extracted from brown algae has been shown to improve aerobic capacity and increase muscle size and strength in mice. Whether these beneficial effects translate to humans is unknown. This study investigated the effect of a resistance training program in combination with fucoidan supplementation on measures of strength and performance in apparently healthy adults. In a double-blind placebo control fashion, 20 participants (9 male and 11 female) were randomised to supplement with fucoidan (*N* = 10, 1 g/day) or placebo (*N* = 10, 1 g/day) during six weeks of resistance training. Body composition, muscle strength, anaerobic performance, and blood measurements were compared pre- and post-resistance training. Strength measured by 1 repetition max back squat was significantly increased in both placebo and fucoidan supplemented groups (*p* < 0.001), with no significant effect of treatment observed (*p* = 0.48). Peak power and relative peak power produced during a Wingate test were significantly increased in the fucoidan supplemented group only (*p* < 0.05). There was a significant treatment effect on body composition (*p* < 0.05), with the fucoidan supplemented group demonstrating a significant increase in lean body mass and a decrease in body fat % (*p* < 0.05). In conclusion, fucoidan supplementation during resistance training may provide a viable strategy for enhancing muscle anaerobic performance as well as improving metabolism and body composition.

## Introduction

Resistance training increases muscle strength and mass and improves body composition to elicit a multitude of metabolic-health benefits and reduced risk of chronic disease^1^. Supplements are commonly used in conjunction with resistance training to enhance performance, optimize muscle growth and fat loss, and support overall fitness goals in a convenient manner^2^. The popularity of global dietary supplements is reflected by the estimated market value of approximately $178 billion in 2023 that is projected to grow at a rate of approximately 9% from 2024 to 2030^3^. With a saturated and profitable market resulting in a growing range of available supplements, rigorous analysis is required to determine their purported effectiveness.

Fucoidan, a bioactive compound derived from various species of brown seaweed, has multiple properties including antioxidant, anti-inflammatory, and anti-obesity effects^4–7^. Recently, fucoidan has received attention for its potential to exert beneficial effects on skeletal muscle health and potential to enhance exercise adaptation and increase muscle strength in animal models^8–12^. Fucoidan extracted from *Laminaria japonica* elicited significantly greater strength and performance measures among mice following a 21-day intervention^9^. A similar positive effect was reported on murine skeletal muscle, where 28 days of supplementation with a blend of fucoidan from *Undaria pinnatifida* and *Fucus vesiculosus* triggered muscle hypertrophy^10^. Furthermore, fucoidan supplementation has been demonstrated to increase gene expression levels of the AMPK/SIRT-1/PGC-1α signalling pathway which is associated with mitochondrial biogenesis^8,12–14^. However, whether these beneficial effects of fucoidan extracts translate to improvements in muscle strength in humans remains unclear.

The purpose of this study was to explore the potential synergistic effects of fucoidan supplementation when combined with structured resistance training. The study aimed to investigate whether the supplementation of a novel blend of fucoidan could enhance the outcomes of resistance training on muscle strength, anaerobic power, and body composition in healthy adults. Based on previous literature, it was hypothesised that supplementation with fucoidan at a dose of 1 g/day will result in a significantly greater increase in muscle strength and anaerobic power, combined with a greater reduction in fat mass compared to a placebo control group.

## Results

All participants in the placebo group completed the full training and post-testing protocols. In the fucoidan group, one participant completed the entire six-week training period and provided a post-trial blood sample. However, they did not complete the one repetition maximum (1RM) back squat and 30 s Wingate Anaerobic Test (WAnT) due to personal circumstances unrelated to the study. As the absence of performance data was due to participant self-withdrawal and not investigator error, and the reason for withdrawal was unlikely to influence body composition or biochemical outcomes, therefore the participant’s data was retained for analyses. This resulted in *N* = 9 for strength and performance measures and *N* = 10 for body composition and blood biomarkers in the fucoidan group.

### Muscle strength

**Fig. 1:**
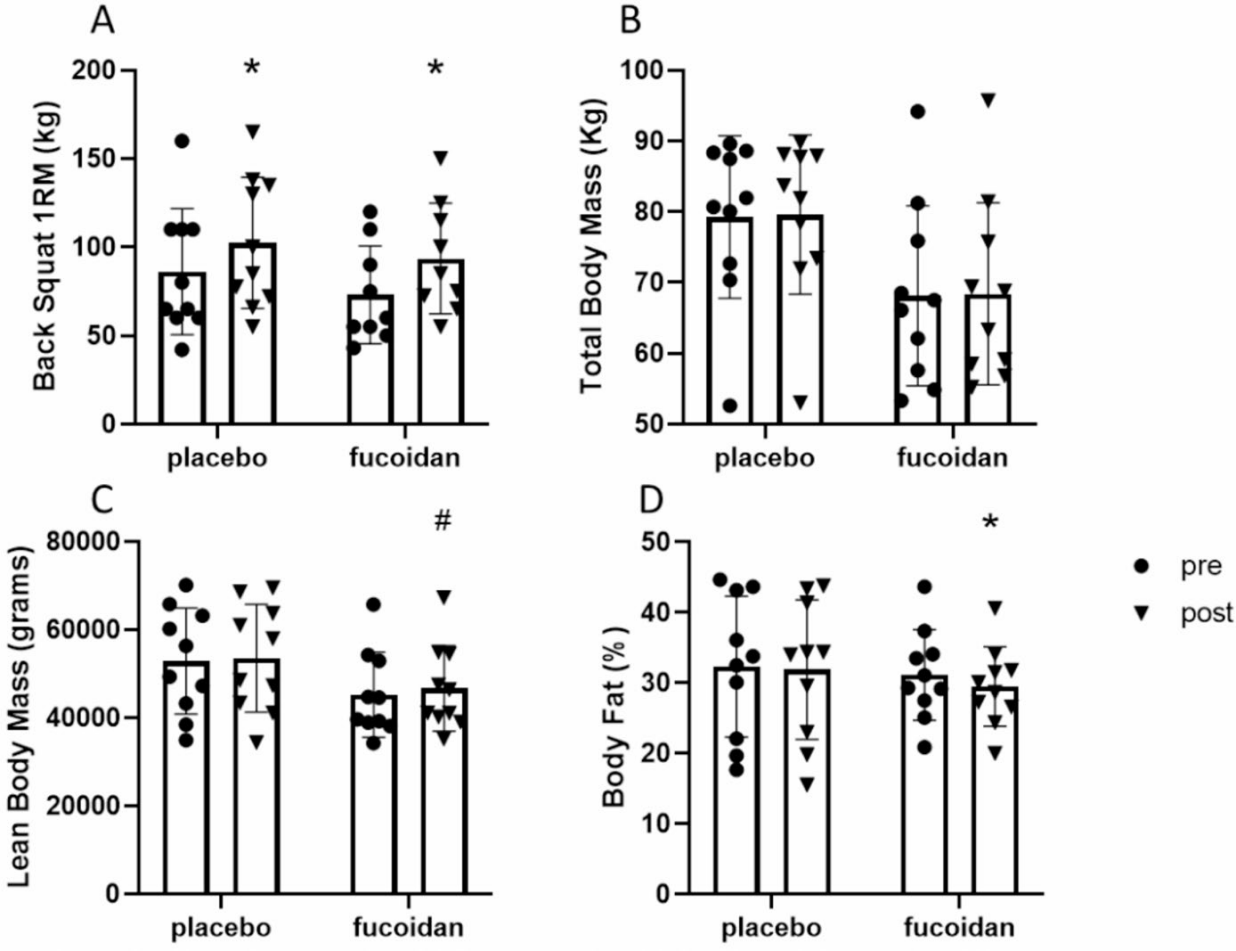
A-D. Changes in muscle strength as measured by back squat one repetition maximum (1RM) (**A**), total body mass (**B**), lean body mass (**C**) and body fat % (**D**) after 6 weeks resistance training in either placebo (*N* = 10 for all measurements) and fucoidan (*N* = 9 for 1RM back squat and *N* = 10 for body composition measurements) supplemented individuals. Filled circles are pre-exercise and filled triangles represent post exercise strength levels. Data are expressed in mean ± SD. * Indicates a significant difference pre-to-post resistance training (*p* < 0.001), # Indicates a significant difference pre-to-post resistance training (*p* < 0.05).

There was a significant effect of our six week resistance training protocol on lower body strength (F_(1,17)_ = 123.1, *p* < 0.0001), with both experimental groups demonstrating increases in strength (placebo: pre-exercise 86.2 ± 35.6 kg, post-exercise 102.4 ± 37.2 kg, *p* < 0.0001, fucoidan: pre-exercise 73.1 ± 27.6 kg, post-exercise 93.6 ± 31.3 kg, *p* < 0.0001; Fig. 1A). Compared to placebo, there was no significant treatment effect of fucoidan on the strength increase post six weeks of resistance training (F_(1,17)_ = 0.51, *p* = 0.48).

## Body composition

Changes in body composition pre- and post-resistance training are shown in Fig. 1B, C and D respectively. There was no effect of resistance training (F_(1, 18)_ = 0.35, *p* = 0.56), supplementation (F_(1, 18)_ = 4.29, *p* = 0.052), or interaction effect (F_(1, 18)_ = 0.01, *p* = 0.92) on total body mass (Fig. 1B). There wasn’t a significant training x supplement (F_(1, 18)_ = 2.19, *p* = 0.16) or a supplement effect (F_(1, 18)_ = 2.17, *p* = 0.16) on lean body mass (LBM). However, there was a significant effect of our 6 week resistance training program on LBM (F_(1, 18)_ = 16.21, *p* = 0.0008), with the post hoc test showing there was a significant difference between pre and post resistance training in the fucoidan supplemented group (*p* = 0.002); Fig. 1C). There was both a resistance training (F_(1, 18)_ = 14.96, *p* = 0.001) and resistance training x supplementation effect (F_(1, 18)_ = 5.22, *p* = 0.03) for body fat percentage (BF%), with the post-hoc analysis identifying a significant decrease between pre and post BF% in the fucoidan treated group (*p* = 0.0008; Fig. 1D); whereas no change occurred in the placebo group (*p* > 0.05).

## Anaerobic performance

**Fig. 2:**
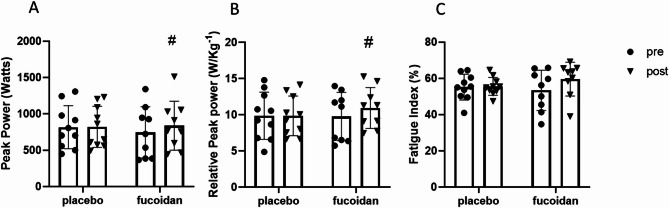
A-C. Changes in anaerobic capacity variables for subjects pre and post six weeks resistance training as measured by peak power (**A**), relative peak power (**B**), and fatigue index (**C**) after 6 weeks resistance training in either placebo (*N* = 10 for all measurements) and fucoidan (*N* = 9) supplemented individuals. Filled circles are pre-exercise and filled triangles represent post exercise strength levels. Data are expressed in mean ± SD # Indicates a significant difference pre-to-post resistance training (*p* < 0.05).

The mixed effects 2-way ANOVA analysis showed that there wasn’t a significant training effect (F_(1,17)_ = 3.98, *p* = 0.068), a significant supplement effect (F_(1,17)_ = 0.04, *p* = 0.78), or a significant interaction effect between resistance training and supplement (F_(1,17)_ = 3.13, *p* = 0.08) on the peak power produced during the Wingate test (Fig. 2A). While there weren’t significant main effects or interactions, the post hoc analysis revealed a significant improvement in peak power in the fucoidan supplemented group (pre- vs. post resistance training, *p* = 0.03) that was not observed in the placebo group. The same outcome was observed for the relative peak power output (Fig. 2B), where there wasn’t a significant training effect (F_(1,17)_ = 3.58, *p* = 0.07), a significant supplement effect (F_(1,17)_ = 0.12, *p* = 0.78, or a significant interaction effect between resistance training and supplement (F_(1,17)_ = 3.84, *p* = 0.06). The post hoc analysis identified a significant improvement in relative anaerobic power output in the fucoidan supplemented group (pre- vs. post resistance training, *p* = 0.03) that was not observed in the placebo group. There was no resistance training (F_(1,17)_ = 2.58, *p* = 0.12), supplementation (F_(1,17)_ = 0.12, *p* = 0.73), or interaction between supplementation and resistance training (F_(1,17)_ = 1.94, *p* = 0.18) effect observed on the fatigue index (Fig. 2C).

## Blood biochemistry

There were no significant resistance training or supplementation effects on circulating HbA1c, cortisol, cholesterol or triglyceride levels as outlined in Table 1 (*p* > 0.05). Adipokine and cytokine responses are presented in Table 2. There was no exercise or treatment effect on circulating levels of any cytokine or adipokine measured.

**Table 1:** Blood parameters after six weeks resistance training supplemented with either placebo or fucoidan.

	Placebo (*N* = 10)		Fucoidan (*N* = 10)	
	PRE-	POST-	PRE-	POST-
**Hba1c (mmol/mol)**	35.9 ± 2.6	35.8 ± 2.7	34.4 ± 3.0	34.2 ± 2.9
**Cortisol (nmol/L)**	470.8 ± 115.8	467.8 ± 132.9	431.7 ± 131.7	400.9 ± 158.2
**Total Chol (mmol/L)**	5.1 ± 0.6	4.9 ± 0.6	4.91 ± 0.6	5.1 ± 0.7
**LDL/HDL ratio**	2.1 ± 0.9	2.1 ± 0.6	1.9 ± 0.8	2.2 ± 0.8
**Triglycerides (mmol/L)**	1.2 ± 0.4	1.4 ± 0.5	1.4 ± 0.7	1.2 ± 0.3

Data are expressed in mean ± SD. Hba1c, haemoglobin A1c, CHOL, total cholesterol; HDL, High-density lipoprotein cholesterol; LDL, Low-density lipoprotein cholesterol.

**Table 2:** Cytokine and adipokine concentrations following 6 weeks of resistance training in placebo or fucoidan treated groups.

	Placebo (*N* = 10)		Fucoidan (*N* = 10)	
	PRE-	POST-	PRE-	POST-
**Adipsin (ng/ml)**	684.47 ± 1868.00	967.04 ± 1998.78	1627.30 ± 1750.25	1193.79 ± 2050.38
**MCP-1 (ng/ml)**	0.62 ± 0.28	0.55 ± 0.21	0.70 ± 0.24	0.72 ± 0.31
**IL-1β (ng/ml)**	0.21 ± 0.34	0.19 ± 0.32	0.11 ± 0.19	0.12 ± 0.19
**IP-10 (ng/ml)**	0.33 ± 0.22	0.29 ± 0.21	0.21 ± 0.16	0.19 ± 0.14
**IL-10 (ng/ml)**	0.29 ± 0.46	0.24 ± 0.39	0.15 ± 0.36	0.15 ± 0.34
**IL-6 (ng/ml)**	0.10 ± 0.19	0.09 ± 0.17	0.05 ± 0.11	0.05 ± 0.11
**IL-8 (ng/ml)**	0.18 ± 0.28	0.15 ± 0.24	0.07 ± 0.11	0.08 ± 0.10
**Leptin (ng/ml)**	7.24 ± 5.57	6.33 ± 5.66	4.18 ± 2.67	4.39 ± 3.56
**IFN** Ɣ **(ng/ml)**	1.79 ± 2.75	1.53 ± 2.45	0.82 ± 1.51	0.82 ± 1.41
**Resistin (ng/ml)**	1.46 ± 0.32	1.42 ± 0.27	1.75 ± 0.42	1.91 ± 0.55
**TNF α (ng/ml)**	0.66 ± 0.98	0.58 ± 0.89	0.29 ± 0.56	0.29 ± 0.56

All cytokines and adipokines are measured in ng/ml and expressed as mean ± SD. MCP-1, Monocyte Chemoattractant protein-1; IL-1β, Interleukin one beta; IP-10, Interferon gamma Induced protein-10; IL-10, IL-6, IL-8, Interleukin 10, 6 and 8 respectively; IFN Ɣ, Interferon gamma; TNF- α, Tumor Necrosis Factor alpha.

## Discussion

This study aimed to investigate whether fucoidan supplementation enhanced resistance training outcomes in healthy adults. As expected, both experimental groups significantly increased lower body muscle strength compared to baseline, while the fucoidan-supplemented group exhibited enhanced power output and fat loss. These outcomes suggest an independent effect of fucoidan supplementation on anaerobic performance and body composition when undertaking a resistance training program.

In the current study, there were no fucoidan mediated improvement in 1RM back squat strength beyond those observed with placebo supplemented resistance training. This is in contrast to previous animal studies that have demonstrated significant improvements in muscle strength^9,10,15^, muscle mass^12^ and fibre size^8^. The discrepancy between human and animal studies on fucoidan’s impact on muscle strength may arise from several variables. Standard mouse models such as C57/Bl6J and BALB/cByJ, have a homogenous genetic background which may not fully capture the genetic variability of human populations^16^. Reported improvements in muscle strength measures in mouse studies is made possible by the utilisation of in situ and in vitro muscle testing protocols which provide accurate assessments of muscle strength. The reduced phenotype variation combined with the more accurate assessment of muscle strength may explain why results from mouse models might not directly translate to human studies. Additionally, there are different species of brown algae which have been shown to influence bioactivity and bioavailability^17^. While there is increasingly favourable data published on the positive therapeutic effect of fucoidan using animal and cell models, the information on its biodistribution and effect in humans is currently insufficient.

In the present study, six weeks of supervised resistance training did not improve peak power during a 30 s Wingate Anaerobic Test (WAnT) in the placebo group. This finding aligns with previous data demonstrating that resistance training does not enhance 30 s WAnT performance in either males^18^ or females^19^. Interestingly, the combination of 1 g/day fucoidan supplementation and resistance training for 6 weeks, increased peak power output and relative peak power output during a 30 s WAnT. This contrasts with earlier studies using the same dose of 1 g/day fucoidan supplementation, which showed that short-term (< 4 weeks) fucoidan supplementation does not influence anaerobic performance following either an acute bout of high-intensity exercise^11^ or three weeks of high-intensity repeated sprint cycle ergometer training^20^. While the absorption, distribution, and metabolism of fucoidan have been confirmed in rat models^21^, very little is known about its bioavailability in humans. Therefore, the dose and duration of supplementation periods should be carefully considered when using fucoidan.

Another possible factor for previous studies not observing an improvement in anaerobic performance may be the species of fucoidan used in comparative studies. Both McFadden and Colleagues^11^, and Cox and colleagues^20^ supplemented participants with a fucoidan extracted from single species (*Undaria pinnatifida*), whereas participants in the current study were supplemented with a blend of (*Undaria pinnatifida* and *Fucus vesiculosus*). It is well established that fucoidans are not all equal, with the bioactive properties of fucoidan being dependent on the extraction techniques, brown algae species of origin, season of harvest, geographical location, algal maturity and which component of the brown algae the fucoidan was extracted from^17^. Due to a lower molecular weight and higher levels of fucose, *Fucus vesiculosus* has been shown to possess greater bioactivity levels than *Undaria pinnatifida*^22–25^, which may explain why previous studies supplementing with *Undaria pinnatifida* did not see improvements in anaerobic performance in < 4 weeks.

Perhaps the most intriguing result in the present study was the significant increase in LBM and the reduction in BF% observed in the fucoidan supplemented group. Numerous studies have demonstrated that fucoidan inhibits lipid synthesis, reduces lipid accumulation and increases lipid metabolism^26^. In mice fed with a high fat diet, treatment with fucoidan has been shown to reduce weight gain^27^, as well as decrease visceral fat and increase subcutaneous fat ratios^28^. Further, cell culture studies suggest that fucoidan increases lipolysis without inducing adipogenesis^29–31^. More recently it has been suggested that fucoidan mediates its lipid lowering effects by enhancing the expression of lysosomal genes^32^. To the authors’ knowledge, this is the first study using fucoidan supplementation that demonstrates an impact on body composition of human participants. Further study is needed to determine if similar or more pronounced findings are observed in obese, overweight and/or sedentary individuals.

The aim of this study was to determine whether the strength changes observed in animal models, translated to humans. Based on Levene’s tests for homogeneity of variances, the pre-resistance training 1RM back squat strength (*p* = 0.552), age (*p* = 0.773), lean body mass (*p* = 0.54) and BMI (*p* = 0.437) of participants all indicate that the variances between the two groups were homogeneous. The study also used the 1RM test to determine strength improvements which is considered the gold standard for assessing muscle strength in non-laboratory situations and has been shown to be a reliable method for evaluating maximal strength in the age range of participants in this study^33^. However, the study is not without its limitations. Participants were asked to maintain their typical diet for the duration of the study and dietary intake was not controlled. It is possible that differences in energy/nutrition intake could have existed and played a role in the changes in fat mass observed in this study. Future research should aim to standardize caloric intake and monitor appetite to assess the full influence of fucoidan on body composition as well as employing a crossover design to ensure that all participants partake in both placebo and fucoidan supplemented groups. Additionally, while the present study was not designed to analyse sex-specific responses to fucoidan supplementation, it is important to acknowledge that sex may influence physiological adaptations to resistance training and nutritional interventions. To date, there is limited evidence in the literature specifically examining sex-based differences in response to fucoidan in human populations. Given known sex-related differences in physiological and metabolic responses to exercise^34^, future research should consider stratifying outcomes by sex or including sex as a covariate in statistical models. Such analyses may help clarify whether fucoidan exerts differential effects in males and females, particularly in the context of exercise adaptation.

In conclusion, the findings from this study suggest that fucoidan supplementation for six weeks combined with resistance training can improve anaerobic performance and body composition in a sample of recreationally healthy adults. These results indicate the potential of fucoidan as an effective supplement to enhance physical fitness and overall health in active individuals.

## Materials and methods

### Participants

Twenty recreationally active adults (M = 9, F = 11) were recruited to participate in this study, which was conducted according to the Declaration of Helsinki and approved by the Institutional Human Ethics Committee (HEC21399). Participants provided written informed consent and were included if they were 18 years or older, were categorised as Tier 1: Recreationally Active individuals^35^ with at least 150 to 300 min moderate-intensity activity or 75–150 min of vigorous-intensity activity a week, plus muscle-strengthening activities 2 or more days a week and were free of musculoskeletal injuries or clinically significant disease preventing participation in a resistance training protocol. Pre-exercise trial characteristics of participants (5 male, 5 female) randomly assigned to the placebo group were age 38.5 ± 11.6 years, height 171 ± 10.6 cm, weight 79.2 ± 11.5 kg, and body mass index (BMI) 28.4 ± 3.3 kg/cm^2. For the participants (4 male, 6 female) assigned to the fucoidan supplementation group, characteristics were age 38.6 ± 11.5 years, height 173 ± 9.1 cm, weight 68.1 ± 12.7 kg, and BMI 24.6 ± 4.5 kg/cm^2. This study was registered on the Australian New Zealand Clinical Trials Registry (ACTRN 12621001632886p).

## Study design

**Fig. 3:**
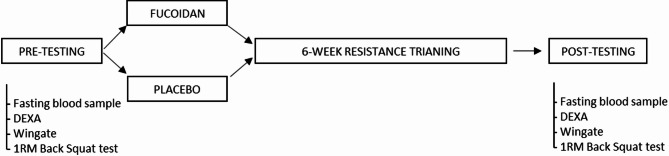
Study design. DEXA, dual x-ray absorptiometry, Wingate anaerobic test, 1RM, 1 repetition maximum. 6-week resistance training consisted of 2 visits each week 2 days apart.

The study was a six-week randomized parallel designed study (Fig. 3). After signing informed consent, eligible participants were assigned a number based on the order in which participants expressed interest. Using the website Research randomizer (https://randomizer.org/), each number was randomly assigned to either group 1 (fucoidan, *N* = 10) or group 2 (placebo, *N* = 10). This process was performed by technical staff that were not involved in the supervision of training program or data analysis. The investigated supplement was 1 g/day of a fucoidan blend combining both *Undaria pinnatifida* and *Fucus vesiculosus* brown algae species (batch # MEF2020560) containing 54.2% neutral carbohydrates, 81.5% fucoidan and 11.2% polyphenols, produced and provided by Marinova (Cambridge, Tasmania, Australia). Participants in the placebo group received 1 g/day of microcrystalline cellulose (GV-MICCEL, Glass Vials, Wetherhill Park, NSW, S, Australia). Participants in both experimental groups were required to take one 500 mg (size zero gelatine capsule) dose in the morning, and a second 500 mg (size zero gelatine capsule) dose in the evening, both with food. Participants were instructed to maintain current dietary habits and refrain from additional resistance training for the duration of the study. All participants reported 100% compliance with the prescribed supplement protocol.

All participants participated in a 6-week resistance program training twice per week with at least 36 h between sessions. This duration/load of resistance training has previously been shown to overcome neural adaptation occurring during the initial 2–4 weeks of a new resistance program^36^, and significantly increase maximal muscle strength in healthy young men^37–39^. Training sessions included two primary exercises alternating between a back squat and deadlift combination and a back squat and bench press combination. These two primary exercises were combined with secondary exercises including lat pulldown/barbell rows, standing shoulder press/lateral raises and a bicep curl/tricep extension superset. Training loads for the compound lifts were based on the pre testing one repetition maximum (1RM) back squat test and estimated 1RM for deadlift and bench press^40^. The training protocol, which included prescribed loads, sets and repetition schemes, was identical for both placebo and fucoidan groups, and was designed using the National Strength and Conditioning Associations, (NSCA) load chart for primary exercises and the repetitions in reserve-based rating of perceived exertion (RPE) scale for the secondary exercises^41^. During the first 2 weeks, the volume was set at 4 sets of 10 repetitions, with loads set at 70% 1RM for primary exercises and an RPE of 5–6 for secondary exercises. During weeks 3 and 4, the volume was set at 4 sets of 8 repetitions, with loads set at 80% 1RM for primary exercises and an RPE of 7–8 for secondary exercises. In the final two weeks of the program, volume was set at 4 sets of 6 repetitions, with loads set at 85% 1RM for primary exercises and an RPE of 8 for secondary exercises. All workouts were supervised by a certified strength and conditioning coach who monitored the training loads and technique^42^. Physical activity/exercise outside of the trial was not monitored, however all training loads were monitored and consistently progressed.

### Body composition assessment

Total body lean mass and fat mass was determined by dual energy x-ray absorptiometry (DEXA; Horizon DXA System, Hologic Inc, USA). Participants were instructed to fast for 8 h prior to the scan, wear minimal clothing free of metal and female participants were screened for pregnancy. A whole-body scan was performed with participants positioned according to manufacturer specifications in a supine position. Body fat percentage (BF%) and lean body mass (LBM) in grams were determined by, and recorded from, the DEXA scan report using Hologic APEX Software (version 5.6.0.1, Hologic Inc, USA).

### Muscle strength

To gain a baseline measure of lower body muscle strength, a 1RM back squat testing protocol was administered after a standardized dynamic warm-up^43^. The test was performed without the aid of lifting material aids and 3–5 min rest was given between attempts. As per the rules of the International Powerlifting Federation (https://www.powerlifting.sport/), to achieve a successful squat attempt, participants had to reach a depth where the hip crease passed below the top of the knee when viewed from the lateral aspect. To begin the protocol, participants performed 8 repetitions at 50% of their estimated 1RM target. This was followed by 3 repetitions at 60% of target 1RM, 2 repetitions at 70% of target 1RM, one repetition at 80% of target 1RM, and by one repetition at 90% of their target 1RM^43^. From this point, attempts were performed to achieve the highest load possible with the Borg scale of RPE score used to determine load increases for subsequent attempts. The final 1RM was recorded if the participant failed to complete the next attempt with an increased load.

### Anaerobic capacity

To assess the anaerobic capacity of participants, a single 30 s Wingate anaerobic test (WAnT) pre- and post- intervention period was performed. This test consisted of a 5 min warmup prior to performing the WAnT which involved pedalling with maximal (all-out) effort for 30 s on an air-braked cycle ergometer (Wattbike Trainer, Nottingham, UK)^44,45^, with the resistance settings determined by the participant’s body weight and sex as per manufacturer’s instructions. Participants began the test with a seated start and received strong verbal encouragement throughout the exercise duration. Measurements of anaerobic performance included: peak power output (PPO, watts), relative peak power output (PPO/body weight, watts/kg), and the fatigue index (peak power/lowest power, %). Participants used the same cycle ergometer pre and post their six weeks of training.

### Blood biochemistry

To assess changes in cytokines^11^ and adipokines^28^ previously reported to be influenced by fucoidan, venous blood samples were collected (following an overnight fast) prior to the pre-trial measurements and within three days after completing the six-week exercise trial. Serum blood markers analysed included glycated haemoglobin (Hba1c, %), cortisol (nmol/L), triglycerides (TG, mmol/mol), total cholesterol (Chol, mmol/mol), high-density lipoprotein cholesterol (HDL, mmol/mol), and low-density lipoprotein cholesterol (LDL, mmol/mol). Blood samples were collected in one ethylenediaminetetraacetic acid (EDTA) containing tube and two serum-separator tubes (SST). One SST and EDTA tube were transported as whole blood sample to a commercial laboratory for immediate analysis (Australian Clinical Labs), with the remaining SST tube centrifuged, serum removed and stored at − 80 °C for future analysis of serum adipokine and cytokine levels. Human adipokines and cytokines (Adipsin, MCP-1, IL-1β, IP-10, IL-10, IL-8, Leptin, IL-6, IFN-γ, Resistin and TNF-α) were multiplexed using a fluorescent bead-based assay (LEGENDplex™ Human Adipokine Panel; BioLegend, San Diego, USA), quantified on a BC CytoFLEX S flow cytometer (Beckman Coulter, USA) and the data was analysed using the LEGENDplexTM Data Analysis Software Suite.

### Statistical analyses

All statistical analyses were conducted using GraphPad Prism v9.1.0 (GraphPad, Boston, MA, USA). To identify a difference in supplementation (placebo or fucoidan), a convenience sample of 10 participants was required (similar in size to previous resistance training interventions^46^ when the alpha (*p* ≤ 0.05) was corrected for repeated measurements and a power of 0.8 (G*Power version 3.1.9.7;, USA)^47^. A Levene’s test for homogeneity of variances was performed on age, BMI and baseline measure of 1RM back squat to assess the cohort homogeneity. A mixed-effects two-way ANOVA was performed to test the effects of our 6-week resistance training protocol and supplementation (placebo or fucoidan). Pairwise comparisons using a Šidāk post-hoc analysis was performed after all two-way ANOVAs to determine differences between pre- and post-6-week resistance training^48^. Statistical difference was set at *p* < 0.05 and all results are presented as mean ± standard deviation (SD).

## Data Availability

The datasets generated and analysed during the current study are not publicly available due to privacy but are available from the corresponding author on reasonable request.
